# Turmeric-Associated Liver Injury: A Rare Case of Drug-Induced Liver Injury

**DOI:** 10.7759/cureus.36978

**Published:** 2023-03-31

**Authors:** David N Smith, Prisca Pungwe, Lauren L Comer, Teminioluwa A Ajayi, Milena G Suarez

**Affiliations:** 1 Gastroenterology and Hepatology, Baylor College of Medicine, Houston, USA

**Keywords:** liver biochemical tests, hepatitis, dili, supplement, turmeric

## Abstract

Turmeric is popularly used as a naturopathic supplement associated with myriad benefits and has long been generally regarded as safe. However, increasing reports of turmeric-associated liver injury have emerged over recent years. This case presents a female patient without significant past medical history who presents with signs and symptoms of acute hepatitis after consuming a turmeric-containing tea. Her case adds to a growing body of evidence that dosage safety, manufacturing, and pharmacologic delivery practices for turmeric supplements should be investigated.

## Introduction

Turmeric is commonly used in cooking, particularly in Asian and Middle Eastern cuisine. It is a member of the ginger family prevalent in South Asia, and its active component curcumin is often marketed to be a safe supplement with many benefits. Turmeric can be consumed in the form of capsules, powders, and teas by patients seeking its potential antioxidant, anti-inflammatory, anti-neoplastic, hepatoprotective, cardioprotective, and neuroprotective benefits [1]. However, hepatotoxicity has been documented as a rare side effect in a few case reports. Here, we present a case of drug-induced liver injury (DILI) in the setting of turmeric supplementation with a resolution of the injury following its cessation.

## Case presentation

**Figure 1:**
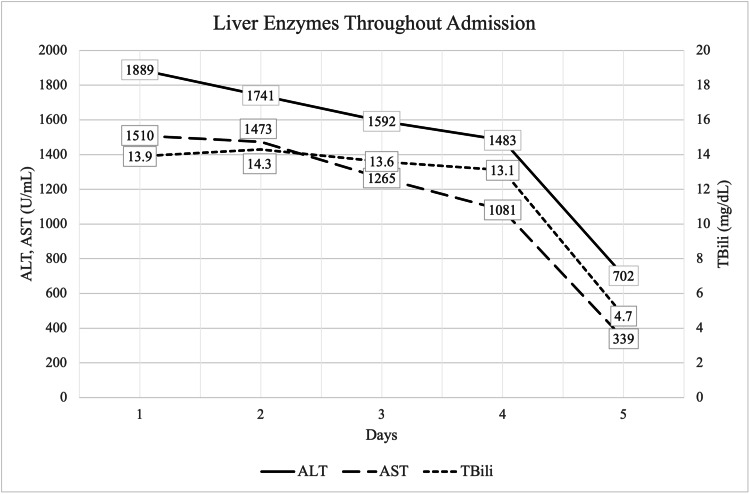
Liver biochemical tests trended throughout the patient’s hospital admission. Alanine transaminase (ALT), Aspartate aminotransferase (AST), Total bilirubin (TBili).

A 62-year-old female with a past medical history of hypertension presented to the emergency department with nausea and generalized abdominal pain for five days. She had no history of hepatic disease or significant alcohol consumption. Her medication history included hydrochlorothiazide and a turmeric tea which she began three weeks prior. Physical exam was notable for scleral icterus and right upper quadrant tenderness. Laboratory workup showed aspartate aminotransferase (AST) 1,510 U/L, alanine aminotransferase (ALT) 1,889 U/L, total bilirubin 13.9 mg/dL, direct bilirubin 8.1 mg/dL, alkaline phosphatase (ALP) 134 U/L, lactate dehydrogenase (LDH) 542 U/L, and an ALT/LDH ratio of 3.49 (Figure 1). Right upper quadrant ultrasound showed centrilobular hyperechogenicity, which can be seen with acute hepatitis. Prothrombin time-international normalized ratio (INR), ferritin, ceruloplasmin, and acetaminophen levels were normal. Alpha-1-antitrypsin, anti-mitochondrial antibodies, and anti-smooth muscle antibodies were negative, as were viral serologies, serum ethanol, and toxicology screen. The patient improved clinically with turmeric cessation, and her liver enzymes returned to normal range upon follow-up after one month.

## Discussion

Turmeric has been used in traditional medicine for centuries, promising numerous potential health benefits, and has become one of the most popular supplements taken over the counter. Total annual retail sales of all herbal supplements in the United States increased by a record-breaking 17.3% in 2020, with turmeric sales grossing nearly 138 million dollars, highlighting rapidly growing national interest in supplement usage [2]. Curcuminoids are turmeric's primary active ingredients. Although their specific mechanisms of action remain unclear, proposed mechanisms include inhibition of cytokine signaling, eicosanoid synthesis, as well as tyrosine kinase, Janus kinase, and cytochrome P450 activity, among many others [1]. Accordingly, the reported therapeutic potential spans a similarly broad range of diseases, although supporting clinical evidence is limited. Effective turmeric use is complicated by low oral bioavailability, rapid metabolism, and chemical instability. Traditional and novel strategies to improve absorption and stability have been investigated, such as co-supplementation with piperine (a major component of black pepper that increases curcumin’s bioavailability by over 2,000%), liposomal encapsulation, nanoparticle complexing, gelation, and others [3]. The Food and Drug Administration approved curcumin for use as an ingredient in various food categories as generally regarded as safe (GRAS), and neither turmeric nor curcumin appears to worsen preexisting liver conditions [4-6]. The few initial reports of adverse responses to turmeric were initially attributed to other precipitating exposures, but suspicion of turmeric-related risk has increased as more reports of turmeric-associated DILI surfaced.

The Roussel Uclaf Causality Assessment Method (RUCAM) is a tool widely used to assess the likelihood of association between a specific medication (in this case, turmeric supplementation) and subsequent liver injury. A score >8 indicates a high likelihood of association, and a score of +9 is the maximum possible score for a young (age < 55), non-alcoholic, non-pregnant patient [7]. This patient’s course of symptom onset, resolution, and lack of concomitant hepatic risk factors garnered a RUCAM score of +9, indicating a high pre-test probability of DILI. As such, a liver biopsy was deemed unnecessary, though pathologic evaluation could be used to underscore the diagnosis. Overall, it is apparent that the underlying pathophysiology and risk factors of turmeric-associated DILI must be closely examined.

Liver injury due to turmeric supplementation appears to follow a dose-dependent framework, with recommendations ranging from 6 to 12 g daily for up to four to 12 weeks [1,4,6,8]. However, this patient’s supplementation in tea obscured the true dosage consumed. Poor recall (for example, regarding the amount consumed or piperine co-supplementation) and unclear product labeling (including specific curcuminoid composition and formulation) further complicated the thorough evaluation of turmeric’s role in this patient’s hepatotoxicity. Other authors have reported possible risk factors, including white race, female sex, and HLA types B*35:01,2 [9]. Lee et al. presented a case of a 55-year-old woman who showed symptoms of liver failure after having a couple of alcoholic beverages [10]. Transaminases were elevated and liver biopsy was significant for interface hepatitis with a mixture of plasma cells, lymphocytes, eosinophils, and neutrophils. Medication reconciliation showed she had been taking a turmeric supplement for three months; following cessation, her symptoms resolved, and her labs returned to baseline. Sohal et al. reported two cases of turmeric-associated DILI with resolution following turmeric cessation [8]. Both patients were women in their 50s who consumed over-the-counter supplements containing turmeric and presented with symptoms of hepatitis and elevated transaminases. Liver biopsies in both patients ultimately revealed hepatocellular injury congruent with DILI, and tests for other causes were normal. Both patients’ symptoms and laboratory values improved within weeks of discontinuing the supplement. Luber et al. documented two cases, one of whom experienced a positive rechallenge with turmeric [11]. She was a 52-year-old woman who co-supplemented turmeric and piperine by capsule for one month (along with occasional diclofenac). She presented with symptoms and biopsy findings consistent with acute hepatitis, initially attributed to diclofenac-associated DILI, which resolved two months after admission. At this point, the patient resumed turmeric supplementation, and her nausea and transaminase elevation returned three weeks later. The patient’s turmeric supplements were negative for contamination with drugs, adulterants, or toxic heavy metals. Her use of the turmeric supplement scored +9 according to RUCAM, and the patient was diagnosed with turmeric-associated DILI.

## Conclusions

The risk of turmeric-associated DILI is often obscured by a general lack of clarity regarding supplement content and dosage, variable pharmacodynamics, and poor patient recall. In the context of growing national interest in patient-direct supplement usage, this case underscores the importance of obtaining a supplement history in cases of DILI and calls for increased transparency and standardization in supplement content, manufacturing, and pharmacologic delivery mechanisms. Furthermore, the examination of maximum safe dosage and specific risk factors for turmeric-associated hepatotoxicity should be revisited.
